# Swabbing as a Non‐Lethal DNA Collection Method for Earthworm Barcoding: Performance and Citizen Science Perspectives

**DOI:** 10.1002/ece3.73385

**Published:** 2026-04-07

**Authors:** Kamyar Amirhosseini, Markus Müller, Martin Potthoff, Oliver Gailing

**Affiliations:** ^1^ Campus Centre of Biodiversity and Sustainable Land Use (CBL) University of Göttingen Göttingen Germany; ^2^ Department of Forest Genetics and Forest Tree Breeding University of Göttingen Göttingen Germany

**Keywords:** COI, DNA barcoding, DNA extraction, Lumbricidae, soil biodiversity, species identification

## Abstract

Lethal specimen collection followed by destructive tissue sampling is employed routinely in earthworm DNA barcoding studies. However, this approach may be ecologically disadvantageous and risks public disengagement with biodiversity and barcoding research due to negative emotional associations, particularly in Citizen Science (CS) projects. The study describes the performance of swab sampling as a non‐lethal and emotionally‐adaptive alternative to tissue sampling in earthworm barcoding. Three DNA collection methods, namely swab sampling with silica‐based DNA extraction (Swab‐QIAGEN), tissue sampling with silica‐based DNA extraction (Tissue‐QIAGEN), and tissue sampling with resin‐based DNA extraction (Tissue‐Chelex) were compared for generating cytochrome c oxidase I (COI) barcodes from 40 adult earthworms. All methods showed comparably high PCR amplification success rates (Tissue‐Chelex = 100%; Tissue‐QIAGEN = 100%; Swab‐QIAGEN = 97.5%), except for one recalcitrant Swab‐QIAGEN sample. Considering the pool of successfully amplified samples, the sequencing success rate for the Tissue‐Chelex, Tissue‐QIAGEN, and Swab‐QIAGEN methods was 82.5% (33/40), 87.50% (35/40), and 66.7% (26/39), respectively—with 25 of 119 barcodes flagged as sequencing/methodological failures due to unusable contigs, stop codons/indels, or contamination. Although swabs showed lower success rates overall, they produced the greatest proportion of reference‐quality barcodes among functional sequences (80.8%; 21/26), indicating their potential for DNA collection compared to Tissue‐Chelex (75.8%; 25/33) and Tissue‐QIAGEN (68.6%; 24/35). All intra‐specimen barcodes, excluding contaminations, received consistent homology‐based taxonomic assignments with average similarity percentage above 99%, corresponding to a 90.4% morphospecies‐genetic data concordance rate. The average intra‐specimen pairwise sequence percentage identity was above 99% (i.e., < 0.1% average sequencing error) across all methods, and no method‐specific clustering was observed in the COI barcode tree. Overall, the results indicated a high degree of interoperability and agreement between swab and tissue sampling methods for recovering the same or nearly‐identical haplotypes with consistent taxonomic identities from each specimen. However, the swabbing methodology requires standardization before it can be confidently utilized in CS‐based earthworm DNA barcoding projects.

## Graphical abstract

Earthworm DNA barcoding commonly relies on lethal tissue sampling, which can discourage participation in citizen science projects due to emotional concerns. By comparing swab‐based and tissue‐based DNA collection methods, we show that swab sampling represents a promising, participant‐friendly option for citizen science–based earthworm biodiversity monitoring, provided that protocols are further standardized.

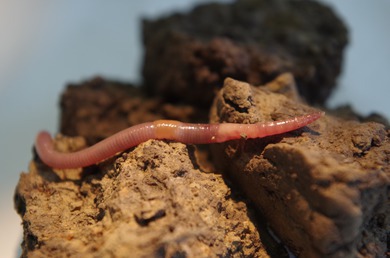

## 1 Introduction

Earthworms hold a well‐recognized and multifaceted significance in soil ecosystem functioning and health (Vidal et al. 2025). They are highly abundant and taxonomically diverse, and influence terrestrial ecosystems through physical, chemical, and biological means (Fierer 2019). Reports of earthworms' global biogeographical patterns predict pronounced changes in earthworm communities and their ecosystem services under future climatic changes and continued anthropogenic pressure, highlighting the necessity of incorporating earthworms into biodiversity research (Phillips et al. 2019). Despite their importance, earthworms are under‐represented in European conservation policies (Köninger et al. 2022). This neglect may be an inadvertent consequence of limited societal connection to soil life (Ristok et al. 2024). Therefore, to better align researchers, citizens, and policymakers toward shared biodiversity conservation goals, it is important to recognize and overcome potential obstacles in the public's engagement with biodiversity research and to develop scientific methodologies by embracing the ethical values of citizens.

Citizen Science (CS), where non‐experts participate in some aspects of research, significantly enhances geographical and taxonomic coverage in biodiversity monitoring (Chandler et al. 2017). These public–professional partnerships reinforce the participants' connection with the natural world and instill a sense of stewardship in them (Novacek 2008). Recent CS projects, such as OPAL in the UK, OPVT in France, Bodemdierendagen in the Netherlands, and Waarnemingen in Belgium (Burton and Cameron 2021), have proven reliable for data acquisition in earthworm ecology and population dynamics (Hoeffner et al. 2025). Among the various scientific approaches and tools deployed through CS, DNA barcoding has repeatedly succeeded in public‐contributed research (Harris and Bellino 2013), owing to its straightforward methodology, adaptable workflow, and compelling concept (Henter et al. 2016).

DNA barcoding offers an accurate, rapid, and a potentially cost‐effective tool for biodiversity monitoring (Hebert et al. 2003), particularly under cost‐optimized protocols for species identification purposes (Hajibabaei et al. 2005; Ivanova et al. 2006). The effectiveness of DNA barcoding, however, relies on the completeness of reference barcode libraries (Twyford et al. 2024). CS‐based DNA barcoding projects contribute to barcode libraries by generating and publishing novel DNA barcodes from under‐sampled areas (Marizzi et al. 2018). SoilRise (www.soilrise.eu) is an international earthworm biodiversity monitoring project between five partner countries (Ireland, France, Germany, Austria, and Poland) which combines CS‐based earthworm sampling with DNA barcoding to enhance earthworm data availability and quality at the European scale. It operates through a unique mentor‐based CS research network, within which university researchers and their assistants supervise the earthworm monitoring project and conduct morphological identifications and DNA barcoding, and trained university students mentor citizen scientists in local earthworm sampling campaigns.

Earthworm sampling methods are broadly distinguished as ethological (i.e., physical/chemical stimulation of earthworms to move out of the soil) or excavation methods, commonly followed by hand sorting (Vidal et al. 2025). After collection, specimens are routinely killed by immersion in ethanol and preserved for subsequent destructive tissue sampling in standard earthworm DNA barcoding wet lab workflows (e.g., Huang et al. 2007; Goulpeau et al. 2025). Euthanizing and preserving specimens offers important scientific advantages and may be necessary for certain purposes. For instance, constructing reference barcode libraries requires high‐quality sequences linked to physical vouchers (Steinke and Hanner 2011). Preserving whole specimens offers sufficient high‐quality tissue for generating robust barcodes, chances for multiple re‐sampling of the same specimen over time, and enables future morphology re‐examination (Twyford et al. 2024). This is not feasible with minimally‐intrusive approaches. Moreover, specimen preservation is critical for studying rare or new species, and taxa (including earthworms) with incomplete libraries (Willows‐Munro and Schoeman 2015), particularly from under‐sampled geographies and biodiversity hotspots, such as the tropics (Oliveira et al. 2016). However, this lethal and destructive approach to collecting genetic material, while DNA‐friendly (de Waard et al. 2008), risks disturbing small, rare, endangered, and declining populations (Régnier et al. 2010; Leung 2024). Considering their role as ecosystem engineers (Blouin et al. 2013), removing earthworms from their habitats may adversely affect the local ecology when conducted at high intensity. Although the removal of small numbers of specimens for DNA barcoding may be considered low‐impact, repeated or large‐scale sampling campaigns, especially from biodiversity‐poor fields or small plots, may reduce earthworm abundance and negatively alter soil functioning (Schon et al. 2021). Destructive tissue sampling also alters the phenotype of voucher specimens (Minamiya et al. 2011; Vin et al. 2011). This can be particularly concerning when multiple samples need to be collected from small specimens, for example during repeated re‐sequencing attempts. While temporally‐spaced resampling of the same specimen might not be possible with certain minimally‐intrusive approaches, particularly those which release the specimens after sampling, on‐field concurrent collection of multiple independent non‐intrusive samples from the same live specimen could, to some extent, resolve the resampling limitation of such approaches. Furthermore, lethal animal sampling is particularly counterintuitive in CS‐based biodiversity monitoring projects, where emotional response and compassion for animals are shown to influence willingness to participate (Greving and Kimmerle 2021; Lin et al. 2024).

Studies on developing non‐invasive DNA sampling methods appear to be taxonomically biased toward vertebrates, and rarely considered for earthworms (Lefort et al. 2022). The few studies available focus on coelomic fluid as a non‐destructive source of DNA in these organisms (Minamiya et al. 2011; Rorat et al. 2014). While the efficacy of non‐destructive surface swabs for DNA collection has been assessed for other terrestrial invertebrates (e.g., slugs; Morinha et al. 2014), it remains to be tested for earthworms. Here, we evaluate the performance of a commercially available swabbing procedure for earthworm barcoding and compare it against two standard tissue‐based methods. Developing a non‐lethal and emotionally adaptive DNA collection method is an important consideration within the context of CS, including the SoilRise project, as it can remove the emotional barrier to lethal/destructive sampling to enhance participation willingness.

## 2 Materials and Methods

### 2.1 Specimen Collection and Sample Preparation

**FIGURE 1:**
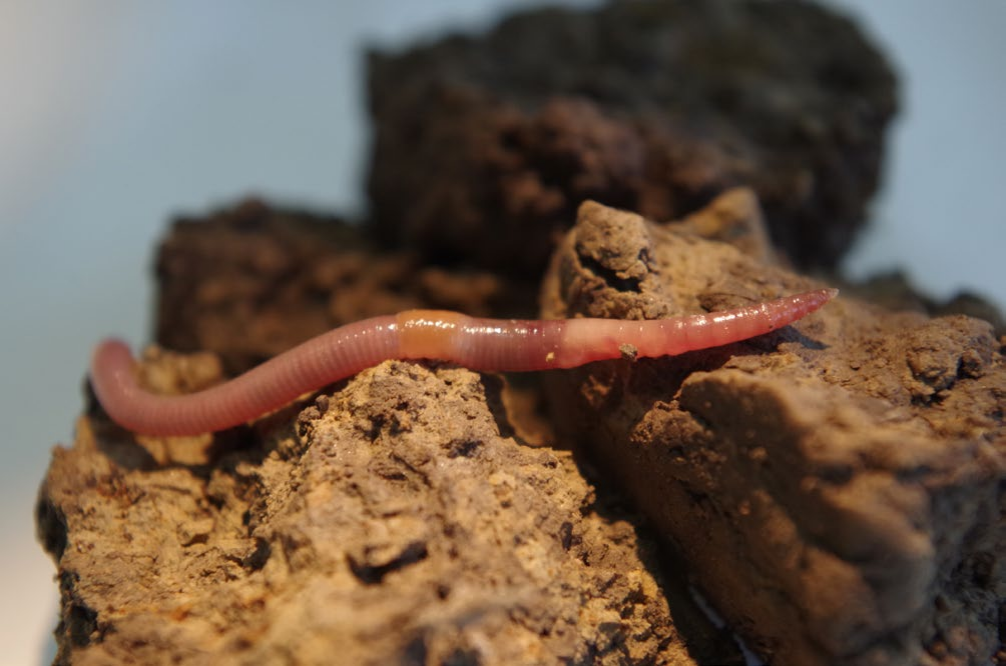
Earthworm specimen. Photographed by the SoilRise team (www.soilrise.eu).

Specimen collection for this study was carried out by mentor‐led citizen scientists as part of the SoilRise earthworm sampling campaign (www.soilrise.eu). In total, 40 adult earthworms (Figure 1) were collected from soil blocks (20 × 20 cm^2^ area to a depth of 25 cm) using hand sorting, from contrasting habitats (e.g., arable fields, vegetable patches or flower beds, and grasslands) across various localities in Lower Saxony and Bavaria (Germany), in October and November 2024. Swab samples (one per each individual) were collected on‐field by swabbing the anterior surface of each live specimen using conical micro‐brushes (Brush Sticks, Dontodent, Karlsruhe, Germany) and placing the brushes in 2‐mL tubes containing a proprietary buffer solution (Sinsoma GmbH, Völs, Austria). To compare and validate the swab‐derived DNA, tissue samples (one per each individual) were also collected from the specimens. To this end, the specimens were transferred into separate 50 mL‐vials containing ethanol (ROTISOLV ≥ 99.9%, HPLC Gradient Grade, Carl ROTH, Karlsruhe, Germany) after swabbing and transported to the lab. No further swab samples were re‐collected from the specimens after preserving them in ethanol. In contrast, tissue samples were re‐snipped from the preserved earthworms throughout the study when necessary—e.g., to resolve a potential lab swab/cross contamination (see the *Genetic data‐morphospecies concordance* subsection of the results). Tissue samples were prepared by researchers in the laboratory by clipping approximately 4 mm^2^ pieces from the specimens' posterior using ethanol‐ and flame‐sterilized scalpels and tweezers. All specimens were visually examined using a stereomicroscope (Digitalmikroskop, Di‐Li, Kaiserslautern, Germany) and morphologically identified by trained university students following established earthworm taxonomic keys. The study primarily uses the identification key by Sherlock (2012) to assign the morphospecies. Additionally, following recent Lumbricidae literature (Pérez‐Losada et al. 2009; Fernández et al. 2012; Csuzdi et al. 2025), this study considered the earthworm species *Aporrectodea caliginosa* and *Aporrectodea trapezoides* as valid species. Following this taxonomic treatment, a secondary key (Reynolds 2022) was consulted to differentiate between these *Aporrectodea* species, as the key by Sherlock (2012) does not consider *A. trapezoides* as a valid species.

### 2.2 DNA Barcoding Workflow

#### 2.2.1 DNA Collection Methods

Two main sampling approaches, namely swabbing and tissue sampling, were compared for their performance in generating functional DNA barcodes for earthworm species identification. For all specimens, DNA from a tissue and a swab sample was extracted using the QIAGEN DNeasy Blood and Tissue Kit (QIAGEN, Hilden, Germany) to enable comparison of the collection methods (tissue snips vs. swabbing) without extraction kit bias. DNA from a second tissue sample was additionally extracted from each specimen using the Chelex 100 Chelating Resin (Bio‐Rad Laboratories Inc., Hercules, California, USA), which is used as our laboratory's standard extraction protocol. Therefore, three DNA collection methods were attempted for each earthworm: 1. Tissue‐Chelex, 2. Tissue‐QIAGEN, and 3. Swab‐QIAGEN. DNA extraction using the QIAGEN kit followed the manufacturer's protocol, with minor modification of the last elution step, whereby 70 μL H_2_O instead of the kit's buffer was used for elution. For Chelex‐based DNA extraction (Zott 2014), tissue samples were incubated with 300 μL of 10% Chelex and 5 μL Proteinase K (20 mg/mL; IST Innuscreen GmbH, Berlin, Germany) at 57°C overnight on a rotary shaker (700 rpm). Next, Proteinase K was denatured at 95°C (15 min), and the extracts were centrifuged at 13,000 rpm (5 min) before the supernatant was used for subsequent PCR amplification.

#### 2.2.2 Amplification

The Folmer region of the mitochondrial cytochrome c oxidase subunit I (COI) gene, recognized as the universal barcode in the animal kingdom, was amplified using the universal “Folmer” primers (LCO1490: 5′‐GGTCAACAAATCATAAAGATATTGG‐3′, HC02198: 5′‐TAAACTTCAGGGTGACCAAAAAATCA‐3′; Folmer et al. 1994). Amplification was carried out through a polymerase chain reaction (PCR) with a final volume of 14 μL, including 1 μL of extracted DNA (average DNA concentrations: ~119 ng/μL for undiluted Tissue‐Chelex extracts, ~106 ng/μL for undiluted Tissue‐QIAGEN extracts, and ~4 ng/μL for Swab‐QIAGEN extracts; all rounded to the nearest whole number) and 13 μL of PCR reagents. The reaction mixture contained 1.5 μL PCR buffer (0.7 M Tris–HCl, 0.175 M (NH_4_)_2_SO_4_, 0.2% w/v Tween‐20; HOT FIREPol 10× Buffer B2, Solis BioDyne, Tartu, Estonia), 1.5 μL MgCl_2_ (25 mM; Thermo Fisher Scientific Inc., Waltham, Massachusetts, USA), 1 μL dNTPs (2.5 mM each; Thermo Fisher Scientific Inc.), 0.2 μL Hot Start Taq Polymerase (5 U/μL; HOT FIREPol, Solis BioDyne), 6.8 μL DNA‐grade H_2_O, and 1 μL of each forward and reverse primers (5 pmol/μL each; synthesized by Sigma‐Aldrich, Darmstadt, Germany). Thermocycler (TProfessional Thermocylcer 96 Gradient, Biometra, Jena, Germany) settings were adjusted to include an initial denaturation at 95°C (15 min) and 35 cycles of denaturation at 94°C (1 min), annealing at 50°C (1 min), and extension at 72°C (1 min), with a final extension at 72°C (20 min), before pausing at 16°C. Amplicons were monitored by running a 1% agarose gel electrophoresis and stained with ROTI GelStain (0.05 μL/mL of gel; Carl ROTH). Each well in the gel held 3 μL of amplicon and 2 μL of 0.25% bromophenol blue loading dye, with 3 μL of 100 bp DNA ladder (GeneRuler, Thermo Fisher Scientific Inc.) pipetted into the first lane.

#### 2.2.3 Sequencing

Enzymatic clean‐up of successful amplicons, as indicated by a positive gel electrophoresis result, was performed by reacting 6 μL of the amplicon with 0.5 μL Exonuclease I (20 U/μL; Thermo Fisher Scientific Inc.) and 1 μL Alkaline Phosphatase (1 U/μL; Fast AP, Thermo Fisher Scientific Inc.). The reaction was incubated first at 37°C (15 min) and then at 85°C (15 min), before pausing at 16°C. The cleaned amplicons were bidirectionally sequenced using the BrilliantDye Terminator (v3.1) Cycle Sequencing Kit (NimaGen, Nijmegen, Netherlands). A cycle sequencing reaction (final volume 10 μL) was set up for each primer, containing 3 μL cleaned amplicon and 7 μL sequencing reaction mixture (including 0.5 μL BrilliantDye Terminator, 2 μL 5× sequencing buffer, 1 μL primer (5 pmol/μL), and 3.5 μL DNA‐grade H_2_O). The cycling profile included an initial denaturation at 96°C (1 min), followed by 35 cycles of 96°C (10 s), 45°C (10 s), and 60°C (4 min), before pausing at 16°C. Sequencing samples were purified using the QIAGEN DyeEx 96 Kit, following the manufacturer's protocol. All sequencing was performed on the Applied Biosystems 3500 xL Genetic Analyzer (Thermo Fisher Scientific Inc.). Sequencing data were collected using Applied Biosystems 3500 Series Data Collection Software v3 (Thermo Fisher Scientific Inc.) and raw sequence data were visualized and checked using Applied Biosystems Sequencing Analysis Software v7 (Thermo Fisher Scientific Inc.). The resulting traces (.ab1 files) were assembled into contigs, employing a quality‐based consensus method in CodonCode Aligner (v12.0.2, CodonCode Corporation, Dedham, Massachusetts, USA).

Amplicons that failed to generate a usable contig on the first attempt (e.g., due to insufficient bidirectional strand similarity) or that received non‐earthworm putative identifications based on homology during contamination screening were re‐sequenced using the same amplification and sequencing protocol. If re‐sequencing did not resolve the issue, the amplification and sequencing protocols were modified slightly to recover the target barcode. These modifications included using 1:10 diluted templates or adding less amplicon (2 μL instead of 3 μL) in the cycle sequencing reactions, particularly for tissue‐extracted samples which yielded higher DNA concentrations compared to swab samples (Appendix 1). Additionally, where non‐specific amplification was suspected, a subset of the samples was subjected to gradient PCR to verify the optimal annealing temperature for specific amplification.

### 2.3 Bioinformatics Workflow

#### 2.3.1 Trace File Pre‐Processing, Sequence Assembly, and Contig Editing

Primer sequences were removed using the vector trimming feature in CodonCode Aligner, and end‐clipping of low‐quality regions was performed to ensure a cleaner and more robust assembly. To increase the usable sequence length while maintaining accuracy, both overlapping and reliable non‐overlapping regions were used to build the consensus. Traces that failed to assemble into usable contigs were flagged as sequencing/methodological failures. Manual base calling was kept to a minimum and limited to conflict resolutions supported by empirical chromatogram evidence, paying attention to features such as peak shape, peak height, background noise, and base‐specific qualities.

Considering the uniparental inheritance (Wallace and Chalkia 2013), lack of heterozygosity, and assuming the rarity of within‐individual heteroplasmy in earthworm protein‐coding mitochondrial genes (e.g., COI), all remaining ambiguous base calls are expected to result predominantly from technical artifacts, such as low signal quality or high background noise in chromatograms. Therefore, all unresolved ambiguities and unconfirmed heteroplasmies in the COI barcodes were represented conservatively by the code “N” (Carracedo et al. 2000; Hanner 2012). Regardless, where unusual or wrong assignments were encountered (e.g., an unusually low similarity percentage in database homology searches or a disagreement between morphospecies and genetic‐based taxonomic identifications, particularly among closely related species) the sequences were rechecked and ambiguous base calls in potentially diagnostic sites were annotated using IUPAC codes. Further information on handling ambiguous base calls and measures taken to minimize information loss is provided in the Supporting Information.

The R package “coil” (Nugent et al. 2020), which uses profile hidden Markov models (PHMMs) to place sequences into a common COI‐5P reading frame and translates them into amino acids to assess the likelihood of erroneous indels, was utilized to monitor the barcodes for stop codons and indels. Sequences flagged for likely stop codons were re‐assembled using more conservative end‐clipping of the corresponding traces' low‐quality ends to improve bidirectional strands' alignment where trace quality issues were suspected. If this did not resolve the issue, a reference‐guided flagging of potentially erroneous insertions was attempted to rescue the barcodes that otherwise had high‐quality sequences. Any heavy editing and reference‐guided correction of the contigs was strictly avoided. Only when empirical trace data confirmed a likely artifact, the erroneous insertion was deleted from the barcode (see Supporting Information for further methodological details). Barcodes with a large number of ambiguities and many stop codons/indels or those with continued structural anomalies were flagged as failed barcodes.

**FIGURE 2:**
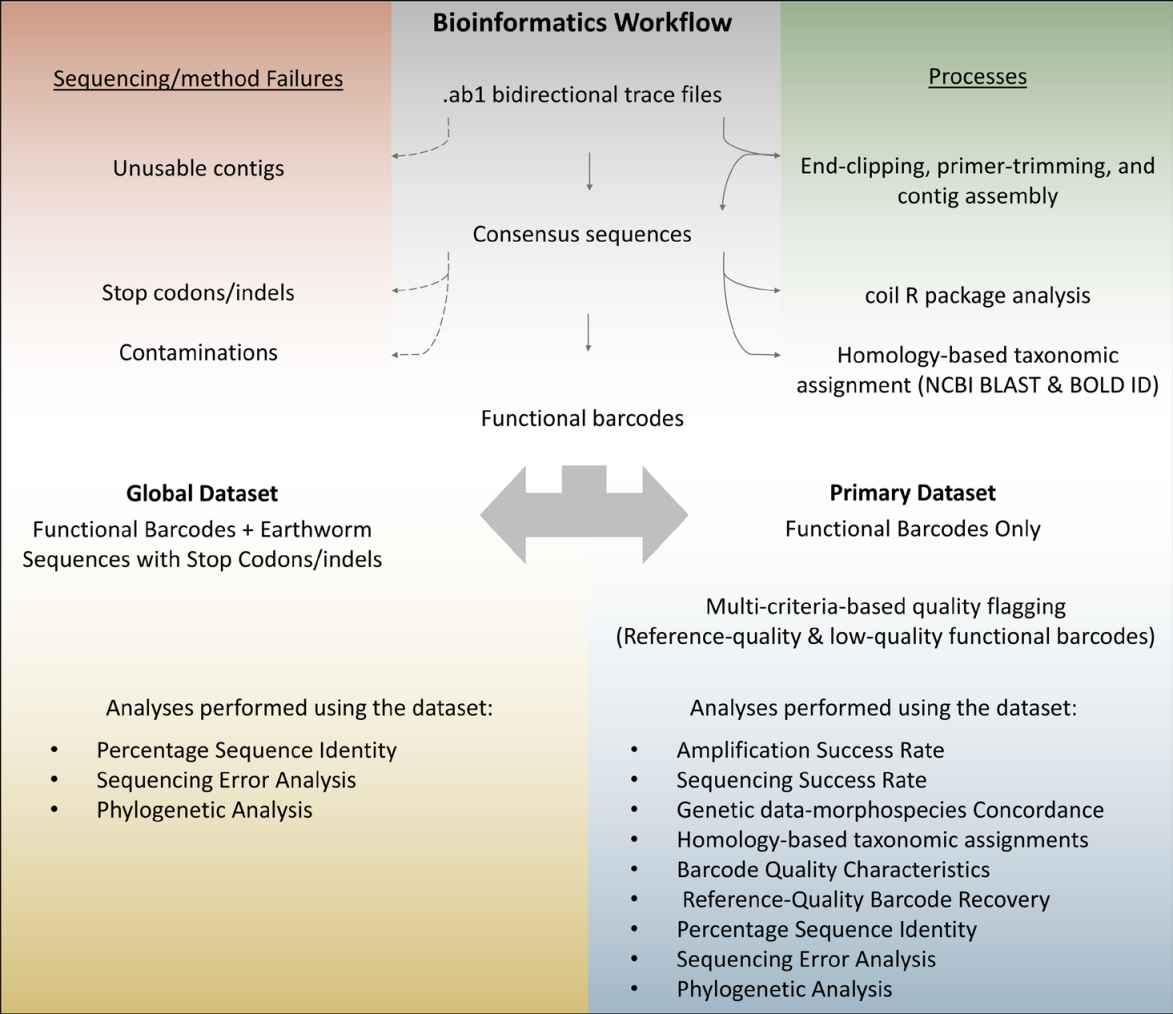
Schematic representation of the study's bioinformatics workflow, showing the sequential steps in generating earthworm barcodes, the datasets used, and the analyses performed using each dataset. Thin arrows represent consecutive processing steps, dashed arrows highlight filtering or exclusion of low‐quality data, and large double‐headed arrows illustrate the difference between the datasets used in parallel.

Moreover, the barcodes were screened for contamination via database homology searches. All usable contigs (i.e., successful assemblies), as well as individual traces from failed contig assemblies, that matched with non‐earthworm taxa (based on NCBI BLAST and BOLD Identification Engine) were flagged as contamination and categorized as sequencing/methodological failures. To sum up, three categories of sequencing/methodological failures were defined and used to filter out non‐functional/failed barcodes from the “primary” dataset: (1) traces with unusable contigs, (2) barcodes with stop codons/indels, and (3) barcodes or traces with non‐earthworm contamination. Therefore, the primary dataset for this study included only functional barcodes not belonging to any of the mentioned sequencing/methodological failure categories. A schematic representation of the study's bioinformatics workflow is provided in Figure 2. Species were assigned by querying the barcodes against NCBI BLAST and BOLD, while considering the taxonomic consistency of the top matches and the confidence metrics of each database (e.g., confidence/similarity percentage, query cover, alignment scores, and E value). When top hits were matched with low confidence (< 97%–98%) or when misidentification was suspected, clustering with reference barcodes (Barraux et al. 2024) was also examined.

All analyses in this study were performed using the primary dataset because it only included functional barcodes. However, to examine methodological differences across the three DNA collection methods under stringent and relaxed criteria and to establish data transparency, a second “global” dataset was also considered. The global dataset included, in addition to all the functional barcodes of the primary dataset, those sequences with internal stop codons/indels (i.e., sequencing/methodological failure category 2), as long as they received earthworm putative taxonomic assignments based on homology. In other words, traces with unusable contigs (i.e., sequencing/methodological failure category 1) and sequences with non‐earthworm contamination (i.e., sequencing/methodological failure category 3) were filtered out of the global dataset. The global dataset was used in parallel to the primary dataset for selected analyses, including percentage sequence identity (PID), sequencing error rates, and barcode clustering analysis, to get an unbiased estimate of non‐functional barcodes (Figure 2). Additionally, the frequency of sequences with non‐earthworm contamination and of traces with unusable contigs are reported for each method.

#### 2.3.2 Barcode Quality Characteristics and Reference‐Quality Barcode Recovery

Functional barcodes of the primary dataset had varying sequence quality characteristics. This quality inconsistency likely hinted that, while all primary dataset sequences are robust for species identification, not all might be reliable as reference barcodes. Therefore, to compare barcode quality differences across the different DNA collection methods within the primary dataset, a set of criteria based on established DNA barcoding bioinformatics (Hanner 2012; Bado 2018; Milton et al. 2023) was used to distinguish between high‐ and low‐quality functional barcodes. Here, we use the term ‘reference‐quality barcodes’ to refer to high‐quality functional barcodes within our dataset conceptually suitable for inclusion in a reference barcode library. Accordingly, a sequence linked to a source earthworm specimen was considered a reference‐quality barcode if, in addition to avoiding all three sequencing/methodological failure categories, it also met all of the following criteria: (1) final contig length longer than 500 bp, (2) ambiguous bases < 1%, (3) non‐N bidirectional coverage (i.e., the percentage of the sequence length covered by overlapping forward and reverse traces, excluding ambiguous bases) across at least 40% of the final contig, (4) contiguous high‐quality bases (Phred score > 20) across more than 75% of the barcode, and (5) a final contig quality score (QS; i.e., average base‐specific quality values) of more than 30. Reference‐quality barcode recovery, then, was calculated as the ratio of reference‐quality barcodes to the total barcodes derived through each DNA collection method. Importantly, these quality criteria were applied only to flag and compare sequence characteristics within the primary dataset and did not create additional sub‐datasets (see Figure 2).

#### 2.3.3 Percentage Sequence Identity, Sequencing Error, and Barcode Clustering Analysis

Percentage sequence identity, sequencing error, and barcode clustering analysis were carried out for both the primary and global datasets, with results of the global dataset reported in the Supporting Information. Sequencing error was computed as pairwise genetic distance between intra‐specimen barcodes, as any variation between the same‐sample sequences was assumed to arise from technical artifacts. Distances were expressed as the observed proportion of differences between sequences (i.e., p‐distance), with the pairwise deletion of gaps/missing data in MEGA 12 (Kumar et al. 2024). Percentage sequence identity was calculated as the complement of the p‐distance, scaled to a percentage (i.e., PID = (1—p‐distance) × 100%). Multiple sequence alignments were constructed using the integrated MUSCLE algorithm (Edgar 2004), with default alignment parameters in MEGA 12. To visualize the barcodes' clustering patterns and evaluate the internal consistency among the extraction methods, a distance‐based clustering analysis was conducted using the Neighbor‐joining method (Saitou and Nei 1987), with distances computed using the p‐distance model and including both transitions and transversions, with pairwise deletion of all ambiguous positions. The statistical reliability of the COI barcode tree was tested using the bootstrap method with 10,000 pseudoreplicates (Felsenstein 1985). Throughout the study, barcodes are labeled and referenced using the initials DE (reflecting the specimens' geographical origin; i.e., Germany) followed by the sample number and the DNA collection method used (e.g., DE 1 Tissue‐Chelex).

## 3 Results

### 3.1 Amplification and Sequencing Success

Of the 120 total amplification attempts (3 DNA sources per each of the 40 earthworm specimens), DNA extracted from one swab sample using the QIAGEN extraction kit (sample DE 44 Swab‐QAIGEN) failed to amplify across all of its three repetitions. The amplification success rates for the Tissue‐Chelex, Tissue‐QIAGEN, and Swab‐QIAGEN DNA collection methods was 100% (40/40), 100% (40/40), and 97.5% (39/40), respectively. After minor amplification/sequencing protocol refinements, re‐sequencing, and post‐sequencing curation of the resulting barcodes, the overall sequencing success rate of the primary dataset was 79.0% (94/119), with 25 cases flagged as complete sequencing/methodological failures (due to unusable contigs, stop codons/indels, or contamination). Considering each method and the pool of samples that successfully amplified, sequencing success rate for the Tissue‐Chelex, Tissue‐QIAGEN, and Swab‐QIAGEN DNA collection methods was 82.5% (33/40), 87.5% (35/40), and 66.7% (26/39), respectively. Table 1 summarizes the sequencing/methodological failure pattern and frequency of non‐functional barcodes across the different methods.

**TABLE 1:** Sequencing/methodological success/failure pattern across different DNA collection methods.

DNA collection methods	Sequencing/methodological failure categories		
	Unusable contig/assembly failure	Unresolved indels/stop codons	Non‐earthworm contamination
Tissue‐Chelex	0% (0/40)	10.0% (4/40)	7.5% (3/40)
Tissue‐QIAGEN	2.5% (1/40)	2.5% (1/40)	7.5% (3/40)
Swab‐QIAGEN	5.1% (2/39)	10.3% (4/39)	17.9% (7/39)

Of the 119 successful amplicons, 11 (6 Tissue‐QIAGEN and 5 Swab‐QIAGEN barcodes) failed to assemble into usable contigs on the first try due to poor‐quality traces with insufficient similarity. Re‐sequencing these samples, without any amplification protocol modifications, rescued four barcodes (1 Tissue‐QIAGEN and 3 Swab‐QIAGEN barcodes). Re‐sequencing with 1:10 template dilution or using less amplicon (2 μL instead of 3 μL) in the BD reactions each rescued two additional sequences, resulting in four rescued Tissue‐QIAGEN barcodes in total (two barcodes rescued from each of the mentioned protocol modifications). The remaining three samples with unusable contigs were QIAGEN‐derived DNA (1 Tissue‐QIAGEN and 2 Swab‐QIAGEN barcodes). Notably, all traces obtained with the Tissue‐Chelex method were successfully assembled into contigs without requiring any additional re‐sequencing.

Overall, 13 barcodes (3 Tissue‐Chelex, 3 Tissue‐QIAGEN, and 7 Swab‐QIAGEN barcodes), spanning seven biological specimens, received non‐earthworm taxonomic assignments based on sequence homology. The gradient PCR results determined 51.1°C as the optimal annealing temperature for specific amplification of the samples, differing slightly from the 50°C used in the original amplification protocol. Adjusting the annealing temperature to 51.1°C or re‐sequencing with and without dilution did not change the outcome for the samples containing non‐target DNA. Three specimens (DE 12–14) consistently yielded barcodes with non‐earthworm assignments across all DNA collection methods. Among these three contaminated samples, all tissue‐extracted DNA was matched with nematode sequences based on homology, while swab‐derived sequences received low‐similarity fungus and insect assignments. The other four swab samples flagged for contamination also covered a wide range of non‐earthworm taxa, including fungi, insects, and mammals (including human sequences).

Coil (R package) analysis of all generated barcodes initially flagged 24 sequences (8 Tissue‐Chelex, 8 Tissue‐QIAGEN, and 8 Swab‐QIAGEN barcodes) likely to have stop codons/indels. These flagged sequences corresponded to 20 specimens, four of which had two flagged barcodes each. Re‐assembly of the flagged sequences with stricter end‐clipping of the low‐quality regions and conservative reference‐guided corrections of potentially erroneous insertions resolved the structural issues in 15 barcodes (4 Tissue‐Chelex, 7 Tissue‐QIAGEN, and 4 Swab‐QIAGEN barcodes), leaving 9 sequences with persistent structural issues that were flagged as failures. These 9 sequences with stop codons/indels were obtained from different samples and were mostly generated using either the Tissue‐Chelex or Swab‐QIAGEN methods. This makes any sample‐specific explanation of the structural anomalies less likely and points to potential method‐specific limitations.

### 3.2 Genetic Data‐Morphospecies Concordance

After excluding the samples with complete sequencing/methodological failures, the primary dataset comprised 94 functional earthworm barcodes (33 Tissue‐Chelex, 35 Tissue‐QIAGEN, and 26 Swab‐QIAGEN barcodes) across 37 biological specimens. Each specimen in the primary dataset was represented by at least one and up to three barcodes. Comparing the homology‐based taxonomic assignments of the genetic data with the morphospecies assigned to the specimens revealed eight contradictions. Morphological re‐examination of the eight specimens by trained university students decisively resolved four disagreements by correcting the initial spurious morphological assignments. Notably, one of these resolved cases pertained to a specimen (DE 17) where the initial morphospecies ( *A. caliginosa* ) contradicted the DNA‐based identification ( *A. trapezoides* ) across all of its barcodes (BLAST: 99.0%; BOLD ID: 100%). The key by Reynolds (2022) was consulted to confidently assign the specimen to *A. trapezoides* . In our study, the genetically identified *A. trapezoides* barcodes from specimen DE 17 formed a well‐supported, coherent sister group to *A. caliginosa* barcodes (see barcode clustering analysis result). Additionally, all of this specimen's barcodes (DE 17 Tissue‐Chelex and DE 17 Swab‐QIAGEN) clustered into the same multi‐species Barcode Index Number (BIN) on BOLD (BIN URI:AAC5530), with the same level of similarity to the other *Aporrectodea* species in the BIN, such as *A. caliginosa* and *Aporrectodea longa* .

Another genetic data‐morphospecies discordance was corrected after morphological re‐evaluation by detecting potential cross‐contamination in the affected sample. The specimen DE 1 was initially represented by two high‐quality, tissue‐extracted barcodes, with the corresponding swab sample flagged as non‐earthworm contamination. The intra‐specimen barcodes DE 1 Tissue‐Chelex and DE 1 Tissue‐QIAGEN were contradictorily identified genetically as *Aporrectodea rosea* (BLAST and BOLD: 100%) and *A. caliginosa* (BLAST: 99.34%, BOLD: 99.67%), respectively. Both the initial morphospecies assignment and morphological re‐evaluation of the discordant specimen confidently identified DE 1 as *A. caliginosa* . As a result, the barcode putatively identified as *A. rosea* (DE 1 Tissue‐Chelex barcode) was suspected as a potential sporadic laboratory cross‐contamination. Re‐extracting and re‐sequencing the affected sample using the Tissue‐Chelex method resolved the discrepancy caused by cross‐contamination, generating a functional *A. caliginosa* barcode (BLAST and BOLD: 99.47%) linked to the source specimen. Overall, morphological re‐examination and control for cross‐contamination reduced genetic data‐morphospecies discordances to three specimens (with three barcodes each), resulting in 91.9% (34/37) overall sample‐level and 90.4% (85/94) barcode‐level concordance between genetic data and morphospecies. Considering each method and the pool of functional barcodes without sequencing/methodological failures, the genetic data‐morphospecies concordance rate for the Tissue‐Chelex, Tissue‐QIAGEN, and Swab‐QIAGEN DNA collection methods was 90.9% (30/33), 91.4% (32/35), and 88.5% (23/26), respectively.

The remaining three specimens with discordant genetic and morphological identifications (specimens DE 9, 37, and 40) each yielded three barcodes, one from each DNA collection method. The discordant barcodes were checked for ambiguities in potentially diagnostic sites, and the generic N codes in these sites were replaced with IUPAC codes based on chromatogram inspection. All intra‐specimen barcodes for each of these samples received consistent homology‐based taxonomic identifications across all methods, questioning the reliability of the morphological assignments (Table 2). Database (BOLD and NCBI) homology‐based queries returned high‐confidence (97%–100%) matches to known sequences for DE 9 (e.g., *Octolasion lacteum* CEA) and DE 37 (e.g., *A. rosea* L1), with the subject sequences showing broad geographic distributions. The DE 40 barcode confidently (100%) matched with *Eisenia andrei* from a large BIN (BOLD:AAA8685), with *E. andrei* majority (675/893 sequences) and a small number of *Eisenia fetida* records. Morphological re‐evaluation of the specimens did not resolve the contradictions. Notably, the students expressed difficulty in assigning confident morphospecies to these earthworms.

**TABLE 2:** Morphological and genetic identifications of three earthworm specimens showing discordance across barcodes obtained using three DNA collection methods (Tissue‐Chelex, Tissue‐QIAGEN, and Swab‐QIAGEN).

Specimen ID	Morphospecies	Genetic ID (All barcodes)	%BLAST	%BOLD ID
DE 9	*Octolasion cyaneum* (Savigny 1826)	*Octolasion lacteum* (Örley 1881)	100	100
DE 37	*Murchieona minuscula* (Rosa 1906)	*Aporrectodea rosea* (Savigny 1826)	100	100
DE 40	*Eisenia fetida* (Savigny 1826)	*Eisenia andrei* (Bouché 1972)	100	100

### 3.3 Homology‐Based Taxonomic Assignments

All 94 barcodes across the 37 specimens in the primary dataset received unanimously concordant intra‐specimen taxonomic assignments based on homology searches. Table 3 presents descriptive measures on database homology searches of earthworm barcodes obtained via the different DNA collection methods. Of the 94 queries made across NCBI and BOLD databases, three barcodes (DE 15 Tissue‐Chelex, DE 44 Tissue‐QIAGEN, and DE 39 Swab‐QIAGEN) received similarity percentages lower than the common 97%–98% species‐level identification cut‐off. Ambiguities in potentially diagnostic sites were denoted using specific IUPAC codes but this did not improve the similarity percentages. Despite their low similarity to subject sequences, these barcodes showed congruent taxonomic identities with their corresponding intra‐specimen sequences that received high similarity percentages. Differences in the barcodes' similarity percentages likely reflect method‐specific technical issues, affecting the barcodes' quality and reliability and, in turn, database matching results. Moreover, queries made with two barcodes (DE 39 Swab‐QIAGEN and DE 44 Tissue‐QIAGEN) returned no matches on BOLD. This observation underscores potential barcode quality issues, while also serving as a reminder of BOLD's more stringent data curation and fundamental inter‐database differences between the search algorithms. A closer look at the sequence characteristics of the three barcodes with low similarity percentages collectively revealed quality issues such as high percentage of ambiguous base calls (> 1%) and short length (< 500 bp). Hence, a further quality‐flagging step was carried out to compare the performance of the different DNA collection methods in generating reference‐quality barcodes within the primary dataset.

**TABLE 3:** Similarity percentages from database homology searches of earthworm barcodes obtained via different DNA collection methods.

DNA collection methods	NCBI BLAST				BOLD identification engine			
	*n*	Ave. ± SD	Median	Min—Max	*n*	Ave. ± SD	Median	Min—Max
Tissue‐Chelex	33	99.7 ± 0.7	100	96.3–100	33	99.9 ± 0.3	100	99.0–100
Tissue‐QIAGEN	35	99.5 ± 1.1	99.8	93.6–100	34	99.8 ± 0.4	100	98.1–100
Swab‐QIAGEN	26	99.6 ± 1.3	100	93.7–100	25	99.9 ± 0.2	100	99.0–100

### 3.4 Reference‐Quality Barcode Recovery

Overall (i.e., relative to the starting pool of 40 earthworm specimens), the reference‐quality barcode (> 500 bp, < 1% ambiguous bases, > 40% non‐N bidirectional coverage, > 75% high‐quality bases, and QS > 30) recovery rate for the Tissue‐Chelex, Tissue‐QIAGEN, and Swab‐QIAGEN methods was 62.5% (25/40), 60.0% (24/40), and 52.5% (21/40), respectively. However, when considering only the sequences in the primary dataset (i.e., relative to all functional barcodes generated, excluding sequencing/methodological failures), the Swab‐QIAGEN method showed a reference‐quality barcode recovery rate of 80.8% (21/26), while Tissue‐Chelex and Tissue‐QIAGEN recovered 75.8% (25/33) and 68.6% (24/35), respectively. Detailed descriptions of individual barcode quality characteristics across the different DNA collection methods are available in Table S1.

### 3.5 Percentage Sequence Identity and Sequencing Error

The average intra‐specimen pairwise PIDs for barcodes in the primary dataset were above 99% across all methods, indicating an estimated average sequencing error (1‐PID) of < 1% (Table 4; see Table S2 for PID and sequencing error results based on the global dataset). Calculating the pairwise sequencing error for all intra‐specimen barcodes in the primary dataset highlighted three pairs with non‐zero differences between same‐sample sequences. In all cases, the variations carried minimal biological significance. The intra‐specimen distances most likely resulted from artificial variations caused by sequencing artifacts (e.g., inconsistent base calls, particularly in low‐quality regions) due to method‐specific barcode quality differences. Available empirical evidence further validated technical artifacts as the principal underlying cause of the variation seen in these three cases. For instance, the discordances among each of the three barcode pairs were consistently concentrated in low‐quality chromatogram regions with low‐resolution, double peaks, or high background noise (Table S3 provides further details on the artifacts observed within these sequences).

**TABLE 4:** Pairwise sequencing error among earthworm barcodes obtained via different DNA collection methods in the primary dataset, involving both reference‐ and functional low‐quality barcodes.

DNA collection methods	#comparisons	Sequencing error (%)		
		Ave ± SD	Median	Min–Max
Tissue‐Chelex versus Tissue‐QIAGEN	31	0.07 ± 0.37	0	0–2.12
Tissue‐Chelex versus Swab‐QIAGEN	23	0.06 ± 0.30	0	0–1.48
Tissue‐QIAGEN versus Swab‐QIAGEN	24	0.06 ± 0.29	0	0–1.47

### 3.6 Barcode Clustering Analysis

**FIGURE 3:**
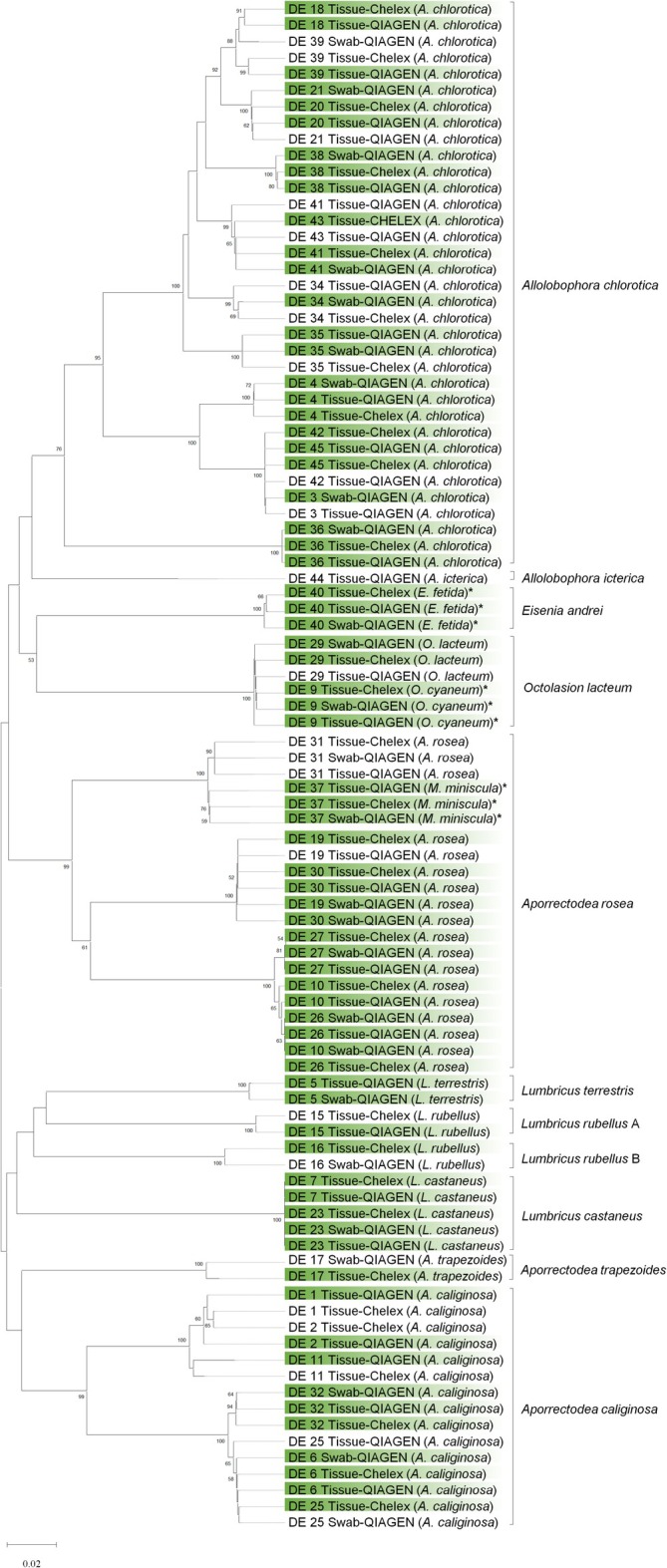
Distance‐based clustering analysis of 94 earthworm barcodes (primary dataset) obtained via different DNA collection methods, using the Neighbor‐joining method with p‐distance as the evolutionary model and pairwise deletion of gaps/missing data, comprising 652 positions and tested with 10,000 bootstrap replicates (values above 50 are shown). External nodes highlighted in green denote barcodes with reference‐quality status. The morphospecies assigned to each sample is shown in parentheses, and asterisks are used to flag barcodes with discordant genetic and morphological identifications. Square brackets show genetic clusters. The letters A and B arbitrarily distinguish the different *L. rubellus* lineages in this study. The COI barcode tree serves a method validation purpose, highlighting the collection methods internal consistency and genetic data‐morphospecies concordance pattern. It does not estimate a species‐level phylogeny.

To maximize the information content on method failure/success pattern, all earthworm COI barcodes in the primary dataset (*n* = 94), regardless of their reference‐quality status, were included in the clustering analysis—excluding only those with sequencing/methodological failure (barcode clustering analysis results of the global dataset are presented in Figure S1). Therefore, the 37 remaining samples are represented on the COI barcode tree by 33 Tissue‐Chelex, 35 Tissue‐QIAGEN, and 26 Swab‐QIAGEN barcodes, with at least one and up to three barcodes per sample from different DNA collection methods (Figure 3). This tree is used for a direct comparison of intra‐specimen barcode clustering consistency and genetic data‐morphospecies concordance only, and not to estimate a species‐level phylogeny. Barcodes with reference‐quality status (designated based on a defined set of criteria) are highlighted in green to indicate the branches that are grounded in high‐quality sequences. Morphospecies assigned to each sample, along with the genetic clustering of the barcodes, are also provided to more clearly present genetic data‐morphospecies concordance patterns.

Method‐specific clustering is absent, confirming that the different DNA collection methods did not bias the barcodes. Most intra‐specimen barcodes cluster together with zero or near‐zero branch lengths in strongly supported clades. For all intra‐specimen barcodes separated by short and near‐zero branches, sample‐wise multiple sequence alignments revealed barcode differences purely due to length differences or infrequent occurrence of ambiguous base calls. This length difference between same‐sample barcodes also explains the interspersed clustering of some conspecific barcodes from different specimens. Few external nodes and sub‐clades, however, warrant attention due to their long branches and/or unusual clustering pattern, reflecting potential biologically meaningful divergence or artificial variations due to technical artifacts.

For instance, while each set of barcodes for samples DE 11 and 39 group together with strong support in higher‐level clades (Bootstrap value = 88–100), their barcodes are separated by long branches. For both DE 11 and 39, sequencing error analysis and chromatogram inspections had established intra‐specimen barcode discordances (Table S3), likely arising from sequence handling artifacts such as ambiguous base calls in low‐quality ends and chromatogram regions with high background noise. Similarly, the intra‐specimen barcodes for DE 17 are separated by a long branch, even though they cluster together into a distinct clade with high bootstrap support (100). The intra‐specimen discordances observed among DE 17 barcodes also displayed signs of technical artifacts, particularly in the sample's Swab‐QIAGEN barcode—for example, discordances in low quality regions. The COI barcode tree recovered all genera as being monophyletic, except for *Aporrectodea*. Figure 3 shows the *A. rosea* clade as separated from the other *Aporrectodea* species and grouped closer to other earthworms. Additionally, barcodes assigned to *Lumbricus rubellus* form two distinct paraphyletic clades, each with high bootstrap support (100).

## 4 Discussion

Barcoding 40 earthworms using the COI marker revealed 10 putative species (11 putative species if the paraphyletic lineages of *L. rubellus* are considered; see genetic clusters in Figure 3). This suggested that the slightly higher number of morphospecies (12) is an overestimation of biodiversity caused by morphological assignment. Additionally, barcoding revealed a taxonomic key limitation by flagging a disagreement between the genetic data, morphospecies, and the available key. This prompted the use of a more comprehensive alternative key to reconcile morphology with the barcode. These observations corroborate the extensive barcoding literature reporting higher identification accuracy using barcoding compared to morphology alone, particularly for cryptic soil organisms with simple phenotypic features, such as earthworms (e.g., Huang et al. 2007; Jänsch et al. 2025). The results also highlight the value of integrating DNA barcoding into CS‐based biodiversity monitoring projects, where morphological assignments by non‐experts can be corroborated by DNA‐based identifications to improve data quality—while acknowledging methodological limitations such as incomplete reference libraries and erroneous database identifications. Such direct performance feedback to the participants plays a vital role in improving identification accuracy and knowledge retention by novices in biodiversity monitoring projects (Van der Wal et al. 2016; Peter et al. 2021). For instance, even in cases where taxonomic keys provide fair diagnostic distinctions using traits that are not inherently similar, non‐expert taxonomists could still face ambiguities, affecting their identification accuracy. Pertaining to the three specimens likely misidentified morphologically (i.e., DE 9, 37, and 40), the identifiers' reported uncertainty in the assignments may have stemmed from ambiguities in subjective interpretations of variable color characters (e.g., when distinguishing between *E. fetida* and *E. andrei* ), difficulty of detecting subtle and low‐contrast character differences (e.g., when differentiating between tubercula pubertatis and marginal thickenings of the clitellum in *A. rosea* and *Murchieona minuscula* diagnostics), and character scoring errors (when depending on precise observations of segment‐based characters in *Octolasion cyaneum* and *O. lacteum* diagnostics). Therefore, barcoding is an important verification and training tool in CS‐based biodiversity monitoring where non‐experts, students, or younger taxonomists conduct the identifications. Similar conclusions were derived by other studies outlining how DNA barcoding enables citizen scientists to accurately identify species and contribute to biodiversity research (Marizzi et al. 2018).

All methods showed comparable PCR success rates, except for one swab‐derived DNA sample that was recalcitrant to amplification in our workflow—potentially hinting at the method's lower sensitivity to recovering sufficient host DNA. However, sequencing/methodological failure counts (Table 1) varied across the methods, with 13 failures in Swab‐QIAGEN, and 7 and 5 in Tissue‐Chelex and Tissue‐QIAGEN, respectively. These observations are consistent with other studies comparing swabs and tissue samples for genetic sampling. For example, several studies report lower DNA yield and higher amplification failure in swabs compared to tissue clips across various terrestrial (e.g., *Acris blanchardi* ; Rainey et al. 2024) and marine taxa (e.g., *Phoxinus phoxinus* ; Macphee et al. 2025). The failure pattern for the three specimens that consistently yielded barcodes assigned to non‐earthworm taxa across all DNA collection methods (i.e., DE 12–14) suggests the issue was unlikely to be method‐specific.

In our study, swabs also recovered a wider range of non‐host DNA (e.g., fungal and human DNA) than did tissue samples. However, fungal assignments should be interpreted cautiously as limited COI amplification success is reported in fungi. Even when successful, fungal COI amplification requires specific primer designs (Dentinger et al. 2011), reflecting the incompatibility of fungal COI with the universal Folmer primers. Moreover, the swab‐derived sequences that received fungal assignments in our study consisted of individual reads with poor quality. Therefore, queries with these sequences may not truly indicate fungal contamination but instead reflect the swab method's vulnerability to low‐quality sequences that risk misidentification or spurious matches. Regardless, these sequences should be considered method failures when comparing the methods' performance. Overall, the contamination screening results indicated that surface swab samples may be more susceptible to environmental contaminations and human DNA carry‐over, requiring more stringent quality control during sampling. Tissue samples, on the other hand, would benefit from careful quality control during gut cleaning procedures. Additionally, preserving the earthworms and preparing tissue samples from them after natural (Messer and Wilkie 2021) or induced depuration (Kandaswamy et al. 2024) could reduce chances of gut biota contamination. Importantly, collecting single swab samples from live specimens prior to ethanol preservation in this study did not allow to obtain fresh swab‐derived DNA extracts when faced with technical errors in processing of the initial sample batch. For this reason, we recommend future non‐intrusive work to concurrently collect multiple independent samples to provide technical buffers, and we posit that having such backup samples may improve swabs' overall performance.

Tissue sampling remains robust for collecting DNA from earthworms. While the Tissue‐QIAGEN method showed 87.5% sequencing success rate compared with 82.5% in Tissue‐Chelex, the Chelex DNA extraction protocol (≈€0.14/sample) was more cost‐effective than the QIAGEN kit (≈€4‐5sample; see Table S4 for further details on cost comparison). The application of the Chelex protocol might considerably reduce costs for large‐scale studies producing a large number of barcodes. In contrast to the tissue‐based methods, the higher proportion of reference‐quality barcodes obtained via the Swab‐QIAGEN method demonstrates that, despite swabs' lower sequencing success rate, this method is able to generate high‐quality barcodes when successful. Here, it is critical to note that our tiered approach to reporting success rates employed thus far (where each rate, such as sequencing error or genetic‐morphospecies concordance, takes into consideration only those samples that survived a preceding analytical step as the denominator of rate calculations) offers an informative look into each method's nuanced weaknesses and strengths that would otherwise be hidden if only global rates were to be considered. But we acknowledge that from an applied citizen science perspective, overall yields also matter. Considering this, the low global success rate of the swabs (23/40 or ≈58%; i.e., from field sampling to accurately identified barcodes in accordance with the morphospecies) currently limits their practical application. Based on these binary outcomes of the Swab‐QIAGEN method—that is, low global success versus high reference‐quality barcode recovery—it may be postulated that a methodological standardization of the initial swab sample collection steps to ensure that sufficient, contaminant‐free host DNA is collected, is needed to enhance swab sampling reliability. Additionally, it is important to note that sequencing failures, rather than taxonomic identity conflicts, primarily limit swabs' readiness for large‐scale adoption. Therefore, attempts at enhancing the sequencing success of swab‐derived DNA, such as collecting multiple swabs per individual or using other sets of host‐specific and/or internal primers, could help improve the method's overall success.

While all citizen scientists were provided with reproducible information on how to collect swab samples from earthworms, the sampling procedure was not monitored individually to ensure consistency, for example, with respect to duration, overall force, or the number of swab strokes. Studies commonly report that protocol optimization regarding various aspects of swab DNA sampling, such as swab type, preservation buffer, and amplification protocol, reduces swabs' failure rates and improves their suitability for large‐scale studies (Martin et al. 2024; Martin and Heathfield 2025). Therefore, swab sampling appears to be a promising method for earthworm DNA collection but might require further standardization for stable utilization.

Table 3 offers a direct comparison of each DNA collection method's performance in generating usable barcodes for species identification. Near‐perfect (> 99%) average similarity percentage across the three methods indicated their high accuracy for earthworm species identification, with variability (min‐max range) suggesting quality differences among the barcodes. Additionally, concordant genetic identifications across intra‐specimen barcodes imply that the different DNA collection methods successfully recovered consistent taxonomic signals for each sample, supporting their technical interoperability. This internal consistency was further validated by the average intra‐specimen pairwise PIDs for barcodes in the primary dataset that were above 99% across all methods. Overall, the tight clustering of same‐sample sequences on the COI barcode tree showed that barcodes from the same organism and different DNA collection methods represent identical or nearly‐identical mitochondrial haplotypes.

The barcode clustering analysis clearly showed that DNA barcoding allows for the distinction between closely‐related and difficult‐to‐diagnose earthworm species, especially when faced with taxonomic key limitations or species complexes. This, for instance, is demonstrated by the DE 17 barcodes (originally misidentified morphologically as *A. caliginosa* ) genetically forming a sister group to the large *A. caliginosa* superclade. The observation is consistent with the *A. caliginosa* species complex taxonomy, in which *A. caliginosa* sensu stricto and *Aporrectodea tuberculata* form a sister clade to *A. trapezoides* , *A. longa* , and *Aporrectodea nocturna* (Pérez‐Losada et al. 2009). Thus, DNA barcoding here enables more reliable species identification than morphology alone, particularly in species complexes and by distinguishing between sister species. Noteworthy, the multi‐species *Aporrectodea* BIN (BOLD:AAC5530)—to which all of sample DE 17 barcodes were assigned—reflects unresolved partitions that warrant expert intervention to resolve the merges. It may further suggest the limited resolution of single‐locus mitochondrial COI barcodes for species delimitation within the *A. caliginosa* species complex and when relationships are complicated by hybridization and introgression (Ratnasingham and Hebert 2013)—as a hybrid origin has been proposed for *A. trapezoides* (Pérez‐Losada et al. 2009). While the COI marker might be efficient for revealing shallow relationships, it alone may not offer sufficient resolution for delimiting very recently‐diverged taxa. Other studies also suggest a multi‐locus DNA taxonomy approach for better discriminatory power in animal species delimitation and identification (Eberle et al. 2020).

**FIGURE A1:**
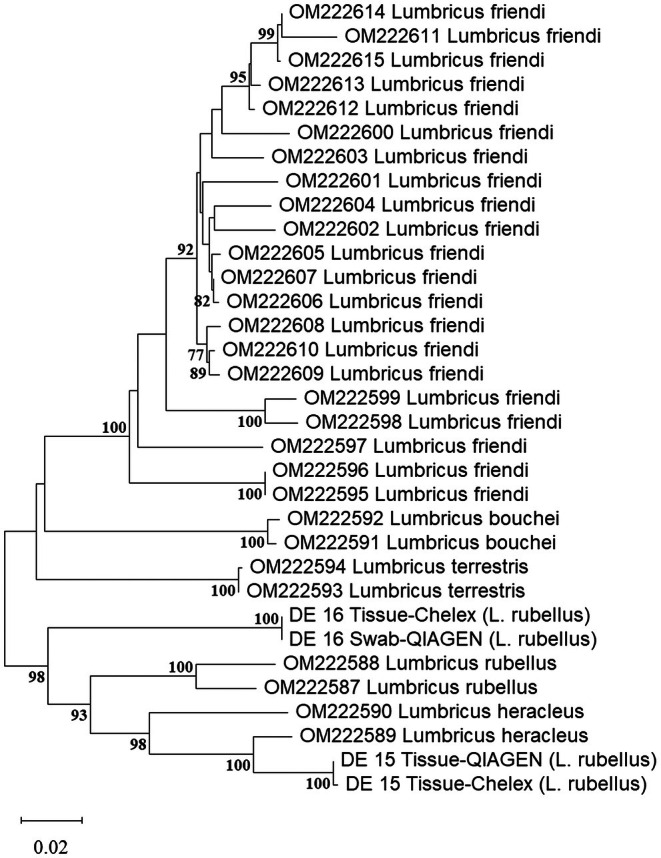
Neighbor‐joining tree of 33 earthworm COI sequences obtained from Briones et al. (2022), including four barcodes generated through this study from the specimens DE 15 and DE 16, using p‐distance as the evolutionary model and pairwise deletion of gaps/missing data, comprising 638 positions and tested with 1000 bootstrap replicates (values above 75 are shown). Barcodes generated through this study are labeled and referenced using the initials DE (reflecting the specimens' geographical origin; i.e., Germany) followed by the sample number and the DNA collection method used (i.e., Tissue‐Chelex, Tissue‐QIAGEN, or Swab‐QIAGEN). The morphospecies assigned to these barcodes are shown in parentheses. The labels for the reference sequences follow those used in Briones et al. (2022). Note that sequences labeled as *Lumbricus heracleus* in that dataset correspond to *Lumbricus friendoides*, following the corrigendum to Briones et al. (2022), which clarified the taxonomic status of these lineages.

The non‐monophyly of the *Aporrectodea* genus in our COI barcode tree, shown by the separation of *A. rosea* from the other species of this genus (Figure 3), is in accordance with other studies (Pérez‐Losada et al. 2012, 2015; Domínguez et al. 2015) that have questioned the validity of the genus using independent mitochondrial and nuclear markers (e.g., COI, COII, 12S, 16S, 18S, 28S, ND1). Such studies report *Aporrectodea* as a non‐monophyletic assemblage, with *A. rosea* appearing as a rogue taxon (Marchán et al. 2023), intermixing with other lumbricids (such as *Octolasion* and *Dendrobaena*). Another interesting observation was the non‐monophyletic grouping of the conspecific samples DE 15 and 16 ( *L. rubellus* ). While still clustered within the larger genus‐level *Lumbricus* superclade, DE 15 and DE 16 share a clade with *Lumbricus terrestris* , making them paraphyletic within our dataset. Database homology searches confidently matched DE 15 and DE 16 barcodes to known deeply divergent lineages within *L. rubellus* (e.g., *L. rubellus* CEA/L2 for DE 15 and *L. rubellus* CEJ for DE 16). Also, the barcodes for these specimens clustered into distinct BINs on BOLD (AAA7354 for DE 15 and AAM1465 for DE 16). Moreover, comparing DE 15 and DE 16 barcodes with COI sequences from a recent *Lumbricus* dataset (Briones et al. 2022) showed DE 15 to cluster with sequences assigned to *Lumbricus friendoides*, and DE 16 as sister to the *L. rubellus* and *L. friendoides* clades (Appendix 2: Figure A1). *Lumbricus friendoides* was recently raised from a subspecies within *L. rubellus* (i.e., *L. rubellus friendoides*) to the species rank (Briones et al. 2022), and the key used in this study does not recognize it as a separate species. As a result, morphology initially placed the specimen within *L. rubellus* , and the genetic data provided additional resolution within the complex. Therefore, the non‐monophyletic grouping of the arbitrarily‐annotated *L. rubellus* lineages A and B in our study (Figure 3) is biologically meaningful and does not indicate methodological inconsistencies. Future work with nuclear markers and integrative taxonomy could further clarify lineage boundaries.

Overall, all DNA collection methods recovered consistent sequences reflecting the specimens' taxonomic identities. Therefore, the interoperability of the considered collection methods can be considered robust for species identification. However, the methods' interchangeability for higher‐resolution applications (e.g., reference library construction) might require further standardization, particularly with respect to swab sampling. The Tissue‐Chelex method remains a reliable and stable approach to generate high‐quality barcodes for both species identification pipelines and reference library construction. The Tissue‐QIAGEN method represents a suitable alternative which has a high amplification and sequencing success rate. However, the quality of the barcodes generated through this method was more variable than of those generated via the Tissue‐Chelex method, often requiring re‐sequencing, slight protocol modifications, or bioinformatics curation and processing. The Swab‐QIAGEN showed to be a promising non‐lethal and non‐destructive method of DNA collection from earthworms. The benign and emotionally‐adaptive nature of this sampling could have an important impact on CS‐based biodiversity monitoring projects, encouraging the participants to partake in sampling campaigns without requiring them to inflict fatalities on the target animal populations. However, swabbing potentially suffers from the lack of a standardization of sample collection, whereby insufficient target DNA acquisition manifests as sporadic contamination or sequences with high background noise limiting downstream analyses. Therefore, the stability and reliability of the swabbing approach to generate usable, high‐quality barcodes depends on careful quality control during initial sampling stages. Future work should focus on optimizing the swabbing protocol by evaluating its key components (e.g., swabbing duration, collection solution, swab type) in relation to barcode quality metrics, amplification and sequencing success, and reference‐quality barcode recovery. Additionally, investigating how swabbing influences participant perception, relative to tissue sampling, would clarify whether swabbing enhances participation willingness in CS earthworm biodiversity monitoring.

## Author Contributions

**Kamyar Amirhosseini:** formal analysis (lead), investigation (lead), visualization (lead), writing – original draft (lead), writing – review and editing (lead). **Markus Müller:** conceptualization (equal), supervision (supporting), writing – review and editing (supporting). **Martin Potthoff:** conceptualization (equal), funding acquisition (lead), resources (equal), supervision (supporting), writing – review and editing (supporting). **Oliver Gailing:** conceptualization (equal), funding acquisition (supporting), resources (equal), supervision (lead), writing – review and editing (lead).

## Funding

This work was supported by Projekt DEAL Open Access funding. Deutsche Forschungsgemeinschaft, 458332906. Biodiversa+, 03LW0510. Niedersächsisches Ministerium für Wissenschaft und Kultur.

## Conflicts of Interest

The authors declare no conflicts of interest.

## Supporting information


**Figure S1:** Distance‐based clustering analysis of 103 earthworm barcodes (global dataset) obtained via different DNA collection methods, using the Neighbor‐joining method with p‐distance as the evolutionary model and pairwise deletion of gaps/missing data, tested with 10,000 bootstrap replicates (values above 50 are shown).
**Table S1:** Barcode quality characteristics across different DNA collection methods.
**Table S2:** Pairwise sequence percentage identity (PID) and sequencing error among earthworm barcodes obtained via different DNA collection methods in the global dataset.
**Table S3:** Pairwise discordances among the three barcode pairs in the primary dataset, based on intra‐specimen sequence differences obtained via different DNA collection methods.
**Table S4:** Cost comparison between the two main DNA extraction protocols used in this study.

## Data Availability

The data that support the findings of this study are openly available on Zenodo at https://doi.org/10.5281/zenodo.17951395 and will be made publicly available upon publication.

## Appendix 1

**Table:**

Sample ID	DNA concentration/NanoDrop reading (ng/μL)		
	Tissue‐Chelex	Tissue‐QIAGEN	Swab‐QIAGEN
DE_1	241.7	92.6	NA
DE_2	216.3	160.4	NA
DE_3	45.9	146.5	NA
DE_4	118.5	158.1	NA
DE_5	228.2	114.0	NA
DE_6	25.9	154.5	NA
DE_7	263.9	127.7	NA
DE_9	192.7	91.3	NA
DE_10	34.5	60.3	NA
DE_11	206.1	122.0	NA
DE_12	211.3	129.7	NA
DE_13	116.9	109.9	NA
DE_14	55.2	116.1	NA
DE_15	13.9	119.9	NA
DE_16	79.0	140.3	NA
DE_17	168.8	122.6	2.1
DE_18	NA	67.6	3.3
DE_19	72.4	77.3	3.0
DE_20	83.5	58.3	3.0
DE_21	248.7	59.9	2.5
DE_23	14.3	144.6	1.8
DE_25	55.5	78.9	2.5
DE_26	NA	52.5	1.6
DE_27	10.3	30.7	3.2
DE_29	79.3	119.8	NA
DE_30	NA	89.0	3.6
DE_31	41.2	35.3	6.2
DE_32	169.9	77.8	6.8
DE_34	173.3	180.9	5.0
DE_35	161.6	103.4	5.5
DE_36	NA	142.4	7.8
DE_37	NA	91.7	2.8
DE_38	37.6	74.8	6.4
DE_39	8.8	83.9	5.2
DE_40	2.7	233.4	4.9
DE_41	41.1	60.8	6.7
DE_42	159.0	141.6	5.7
DE_43	NA	103.1	6.1
DE_44	108.9	133.2	5.1
DE_45	18.9	65.2	4.8
