# Supplementary material for: Swabbing as a Non‐Lethal DNA Collection Method for Earthworm Barcoding: Performance and Citizen Science Perspectives
Source: Ecol Evol. 2026 Apr 7;16(4):e73385. doi: 10.1002/ece3.73385 (PMC13054239; doi:10.1002/ece3.73385)
Supplement: Supplementary file 1 — Figure S1: Distance‐based clustering analysis of 103 earthworm barcodes (global dataset) obtained via different DNA collection methods, using the Neighbor‐joining method with p‐distance as the evolutionary model and pairwise deletion of gaps/missing data, tested with 10,000 bootstrap replicates (values above 50 are shown). Table S1: Barcode quality characteristics across different DNA collection methods. Table S2: Pairwise sequence percentage identity (PID) and sequencing error among earthworm barcodes obtained via different DNA collection methods in the global dataset. Table S3: Pairwise discordances among the three barcode pairs in the primary dataset, based on intra‐specimen sequence differences obtained via different DNA collection methods. Table S4: Cost comparison between the two main DNA extraction protocols used in this study. [file ECE3-16-e73385-s001.docx]

**Supplementary Material**

Supplementary Materials and Methods

*Trace file pre-processing, sequence assembly, and contig editing*

In this study, we used the generic code ‘N’ to denote low-quality, ambiguous base calls in non-diagnostic sites in our barcodes. Using ‘N’ instead of the more-specific IUPAC ambiguity codes ensured compatibility of the genetic data across the study’s bioinformatics workflow and software tools (e.g., between coil and MEGA; see following paragraphs for details). To prevent excessive genetic information loss, the pairwise-deletion option of handling ambiguous bases and gaps was consistently selected in all downstream genetic and barcode clustering analyses. This option, as opposed to the complete-deletion option which removes all ambiguous sites prior to the analysis (Kumar et al., 2024), removes ambiguous positions (N or otherwise) in pairwise comparisons. The pairwise deletion of ambiguity and gaps avoids removal of a large fraction of the sites from consideration (Hall, 2018). Nevertheless, we acknowledge that our approach to annotating ambiguous base calls potentially masks true low-frequency heteroplasmic variants (Parakatselaki & Ladoukakis, 2021). Yet, the reliability of the study’s approach to handling ambiguity is expected to remain uncompromised as the vast majority of the N base calls occurred exclusively in low-quality chromatogram regions, increasing confidence in the assumption that they were technical in origin.

To assess potentially erroneous insertions in barcodes flagged by coil (R package; Nugent et al., 2020) for subsequent deletion from the sequences, separate multiple sequence alignments of the in-house flagged contigs and high-quality conspecific sequences retrieved from the NCBI nucleotide database (via stringent search field descriptions, e.g., scientific name[ORGN] AND COI[GENE] AND BARCODE[KYWD] AND 500:658 [SLEN]) were constructed in MEGA12 (Kumar et al., 2024), using the integrated MUSCLE algorithm (Edgar, 2004), with default alignment parameters. Each resulting MSA was visually inspected for positions where the flagged contig uniquely forced a gap in all other reference sequences. These positions were recognized as likely sequencing or assembly errors and were cross-referenced with the chromatograms for empirical evidence supporting technical artifacts (e.g., bidirectional strands misalignment or ambiguity at the flagged position).

Supplementary Results

*Barcode Quality Characteristics*

Table S1 reports barcode quality characteristics across the different DNA collection methods using descriptive measures. Overall, Tissue-Chelex consistently outperformed the other two collection methods in individual quality metrics. Both Tissue- and Swab-QIAGEN methods commonly displayed higher variability in individual quality metrics compared to those of Tissue-Chelex. Considering all COI sequences in the primary dataset (i.e., reference- and low-quality barcodes), the Swab-QIAGEN method also performed better than the Tissue-QIAGEN method in most individual quality metrics, except for percentage of ambiguities. This better performance of the swab samples was in spite of the method’s higher overall failure rate. Comparing the standard deviation of the quality metrics for reference-quality barcodes with those in the entire primary dataset reveals generally lower variability in barcode characteristics. This lower variability suggests that the criteria used to distinguish reference-quality barcodes successfully excluded marginal sequences. Among the reference-quality barcodes, Tissue-Chelex barcodes maintained a uniform standard of quality and Swab-QIAGEN outperformed Tissue-QIAGEN in most quality metrics, except for contiguous high-quality bases. Both QIAGEN-based methods, however, generally displayed greater variation compared to Tissue-Chelex.

Table S1 Barcode quality characteristics across different DNA collection methods

*Percentage sequence identity and sequencing error (Global dataset)*

The global dataset (n=103) comprised all the functional earthworm barcodes of the primary dataset (n=94) plus 9 more barcodes (4 Tissue-Chelex, 1 Tissue-QIAGEN, and 4 Swab-QIAGEN), that were excluded from the primary dataset because they had stop codons/indels. Despite such structural anomalies, these 9 barcodes received earthworm-related taxonomic assignments based on homology. Notably, all 5 tissue-extracted barcodes with stop codons/indels (4 Tissue-Chelex and 1 Tissue-QIAGEN) received homology-based taxonomic assignments that were congruent with each corresponding specimen’s morphospecies and intra-specimen counterparts—albeit with low similarities (average BLAST similarity percentage=93.3%). Among the 4 Swab-QIAGEN barcodes with structural issues, only one barcode (DE 20 Swab-QIAGEN) received taxonomic assignment (BLAST similarity percentage=97.1%) consistent with the affected specimen’s morphospecies and its other intra-specimen barcodes. The other 3 swab-derived barcodes with stop codons/indels matched with *A. rosea* sequences with low similarities (average BLAST similarity percentage=88.7%), contradicting the samples’ morphospecies and intra-specimen barcodes. These three barcodes also clustered within the *A. rosea* clade with long branches (Figure S1). These observations confirm that COI barcodes with internal stop codons and erroneous indels adversely impact species identification accuracy and reliability. Further, the results infer that structurally-compromised sequences from tissue samples are less sensitive to spurious identifications compared to similar sequences obtained from swab samples. Table S2 summarizes the percentage sequence identity and sequence error among the barcodes in the global dataset. In the global dataset, 11 intra-specimen barcode pairs—in addition to the three pairs flagged in the primary dataset (table S3)—had non-zero sequencing errors, ranging from 0.595% to 23.5%. Evidently, inclusion of sequences with stop codons/indels in the global dataset lowered pairwise PID and inflated pairwise sequencing error compared with the primary dataset results, particularly affecting the comparisons involving swab-extracted barcodes.

Table S2 Pairwise sequence percentage identity (PID) and sequencing error among earthworm barcodes obtained via different DNA collection methods in the global dataset

| **DNA Collection Methods** | **No. of Comparisons** | **PID(%)** | | | **Sequencing Error (%)** | | |
| --- | --- | --- | --- | --- | --- | --- | --- |
|  |  | Ave±SD | Median | Min-Max | Ave±SD | Median | Min-Max |
| Tissue-Chelex vs. Tissue-QIAGEN | 36 | 99.77±0.74 | 100.00 | 96.36-100.00 | 0.23±0.74 | 0.00 | 0.00-3.64 |
| Tissue-Chelex vs. Swab-QIAGEN | 30 | 97.92±5.90 | 100.00 | 76.49-100.00 | 2.08±5.90 | 0.00 | 0.00-23.51 |
| Tissue-QIAGEN vs. Swab-QIAGEN | 29 | 97.93±6.00 | 100.00 | 76.49-100.00 | 2.07±6.00 | 0.00 | 0.00-23.51 |

Table S3 Pairwise discordances among the three barcode pairs in the primary dataset, based on intra-specimen sequence differences obtained via different DNA collection methods

| **Sample** | **Pairwise comparison** | **Sequencing error (%)** | **Supporting empirical evidence** | **Proposed variation explanation** |
| --- | --- | --- | --- | --- |
| DE 11 | Tissue-Chelex vs. Tissue-QIAGEN | 2.12 | Discordances restricted to first 150 bp of the alignment corresponding to regions lacking bidirectional support (Tissue-Chelex) and regions with low-quality/high-noise (Tissue-QIAGEN); in most discordances the non-conflicting base on the Tissue-QIAGEN barcode was overwritten by a conflicting base due to the quality-based consensus method of the assembly algorithm; extensive manual editing was avoided to preserve method-specific failures | Technical artifacts from localized discordances in low-quality regions and algorithm limitations causing false peak detection |
| DE 39 | Tissue-Chelex vs. Swab-QIAGEN | 1.48 | Tissue-Chelex with acceptable quality; discordances concentrated in low-quality ends of the Swab-Qiagen or regions with high background noise | Technical artifacts from base call ambiguity in low-quality regions of the Swab-QIAGEN barcode |
| DE 39 | Tissue-QIAGEN vs. Swab-QIAGEN | 1.47 | High quality Tissue-QIAGEN; 100% identity between tissue-extracted barcodes | Technical artifacts from Swab-QIAGEN barcode quality issues validated by the concordance between other two barcodes |

Barcode Clustering Analysis (Global dataset)

In the COI barcode tree re-constructed using the global dataset, reference-quality barcodes and sequences with stop codons/indels are highlighted with green and red, respectively (Figure S1). The overall topology of the tree remains similar to that of the primary dataset (figure 2). However, incorporating barcodes with structural issues adds some long branches to the tree and results in unusual clustering. For instance, all tissue-extracted barcodes with red highlights group close to their corresponding same-sample barcodes and inside correct species-levels genetic clusters (e.g., DE 3 TissueChelex, DE 5 Tissue-Chelex, DE 16 Tissue-QIAGEN, DE 21 Tissue-Chelex, DE 44 Tissue-Chelex). Conversely, all (DE 7 Swab-QIAGEN, DE 11 Swab-QIAGEN, and DE 42 Swab-QIAGEN) but one Swab-QIAGEN barcode (DE 20 Swab-QIAGEN) are spuriously placed in the *A. rosea* genetic cluster, contradicting these barcodes’ intra-specimen counterparts and the morphospecies assigned to them. These swab-derived sequences mostly failed to recover the full length of the barcode region and were replete with base call ambiguities. The presence of stop codons in the barcodes further reflect their overall low quality. This is especially true for barcodes generated via the swab DNA collection method, as reflected by such barcodes’ misplacement on the COI barcode tree (i.e., DE 7 Swab-QIAGEN, DE 11 Swab-QIAGEN, and DE 42 Swab-QIAGEN).

Figure S1 Distance-based clustering analysis of 103 earthworm barcodes (global dataset) obtained via different DNA collection methods, using the Neighbor-joining method with p-distance as the evolutionary model and pairwise deletion of gaps/missing data, tested with 10000 bootstrap replicates (values above 50 are shown). External nodes highlighted in green denote barcodes with reference-quality status and those in red indicate barcodes with stop codons/indels. Morphospecies assigned to each sample are shown in brackets and asterisks are used to flag barcodes with discordant genetic and morphological identifications. Square brackets show genetic clusters. The letters A and B arbitrarily distinguish the different L. rubellus clades. The COI barcode tree serves a method validation purpose, highlighting the collection methods internal consistency and genetic data-morphospecies concordance pattern. It does not estimate a species-level phylogeny.

*Cost Comparison*

Table S4 summarizes kit-level information and cost comparison for the two main DNA extraction protocols used in our study: a. resin-based Chelex 100 protocol, and b. silica-based QIAGEN DNeasy Blood and Tissue Kit. The calculations consider only the major kit/resin and their components (if any). Prices for Proteinase K (when purchased separately), ethanol, buffers, plasticware, labour, and other consumables or equipment (e.g. pipette tips, gloves, centrifuge, etc.) are not included. For a more thorough comparison, these costs should be considered using laboratory-specific values (For more cost analyses using the Chelex 100 and other commercial kits see: Strøm et al., 2014; Lienhard & Schäffer, 2019; Pacheco et al., 2023). Swabs were collected using conical micro-brushes (Brush Sticks, Dontodent, Karlsruhe, Germany; 150 sticks; €1.95) and placing them in 2-ml tubes containing a proprietary buffer solution (Sinsoma GmbH, Völs, Austria; €2.00 per unit). These were subsequently isolated using the DNeasy Blood & Tissue Kit. Therefore, the cost per sample for the Swab-QIAGEN DNA collection method was about €6.42 (≈€0.01 per Brush Stick + €2.00 per collection buffer unit + €4.41 (Account pricing; see Table S4 caption) per QIAGEN extraction kit preparation).

Table S4 Cost comparison between the two main DNA extraction protocols used in this study.

| **Kit/Protocol Information** | **DNeasy Blood & Tissue Kit (50-prep)** | **Chelex 100 Chelating Resin, (50 g)** |
| --- | --- | --- |
| Manufacturer | QIAGEN | BIORAD |
| Catalogue number | 69504 | 1421253 |
| Kit format | columns | resins |
| Unit price (€; Account pricing^*^) | 207 | 239.70 |
| Unit price (€; List price^**^) | 224 | 282 |
| Samples per unit | 50 samples | ≈1667 samples^†^ |
| Cost per sample (€; Account pricing) | 4.14 | ≈0.14 |
| Cost per sample (€; List price) | 4.48 | ≈0.17 |

*Account pricing refers to the prices (excluding all applicable taxes) actually paid through the university’s customer account. **List price corresponds to the current vendor list prices (excluding all applicable taxes) reported by the manufacturers (reference year: December 2025). †Assuming that Chelex 100 is prepared as a 10% (w/v) suspension, and each extraction uses 300 µL of this suspension (i.e., 0.03 g Chelex per extraction).

Supplementary References

Edgar, R. C. (2004). MUSCLE: multiple sequence alignment with high accuracy and high throughput. *Nucleic Acids Research*, *32*(5), 1792–1797.

Hall, B. G. (2018). *Phylogenetic trees made easy: A how-to manual* (5th ed.). Oxford University Press. https://lccn.loc.gov/2017026596

Kumar, S., Stecher, G., Suleski, M., Sanderford, M., Sharma, S., & Tamura, K. (2024). MEGA12: Molecular Evolutionary Genetic Analysis version 12 for adaptive and green computing. *Molecular Biology and Evolution*, *41*(12), msae263.

Lienhard, A., & Schäffer, S. (2019). Extracting the invisible: Obtaining high quality DNA is a challenging task in small arthropods. *PeerJ*, *7*, e6753.

Nugent, C. M., Elliott, T. A., Ratnasingham, S., & Adamowicz, S. J. (2020). Coil: An R package for cytochrome c oxidase I (COI) DNA barcode data cleaning, translation, and error evaluation. *Genome*, *63*(6), 291–305. https://doi.org/10.1139/GEN-2019-0206/SUPPL_FILE/GEN-2019-0206SUPPLA.ZIP

Pacheco, J. I. M., dos Anjos, K. B. A., Silva, I. V., Okar, R. G., Rodrigues, S. M. B. D., Francabandiera, A. I., & Rodriguez, M. C. (2023). Comparison of two affordable DNA extraction methods for molecular detection of Salmonella isolates from broiler farm’s boot swabs. *Research, Society and Development*, *12*(1), e28312139618–e28312139618.

Parakatselaki, M.-E., & Ladoukakis, E. D. (2021). mtDNA heteroplasmy: Origin, detection, significance, and evolutionary consequences. *Life*, *11*(7), 633.

Strøm, G. E., Tellevik, M. G., Hanevik, K., Langeland, N., & Blomberg, B. (2014). Comparison of four methods for extracting DNA from dried blood on filter paper for PCR targeting the mitochondrial Plasmodium genome. *Transactions of the Royal Society of Tropical Medicine and Hygiene*, *108*(8), 488–494.
